# Plitidepsin as a successful rescue treatment for prolonged viral SARS-CoV-2 replication in a patient with previous anti-CD20 monoclonal antibody-mediated B cell depletion and chronic lymphocytic leukemia

**DOI:** 10.1186/s13045-021-01220-0

**Published:** 2022-01-10

**Authors:** P. Guisado-Vasco, M. M. Carralón-González, J. Aguareles-Gorines, E. M. Martí-Ballesteros, M. D. Sánchez-Manzano, D. Carnevali-Ruiz, M. García-Coca, R. Barrena-Puertas, R. García de Viedma, J. M. Luque-Pinilla, G. Sotres-Fernandez, J. M. Fernández-Sousa, X. E. Luepke-Estefan, J. A. López-Martín, J. M. Jimeno

**Affiliations:** 1 Department of Internal Medicine, Hospital Universitario Quironsalud Madrid, Madrid, Spain; 2 Universidad Europea, Madrid, Spain; 3 Research and Clinical Trials Unit, Hospital Universitario Quironsalud Madrid, Pozuelo de Alarcón, Madrid, Spain; 4 Department of Hematology, Hospital Universitario Quironsalud Madrid, Madrid, Spain; 5 Department of Microbiology, Hospital Universitario Quironsalud Madrid, Madrid, Spain; 6 Pharmamar, Colmenar Viejo, Madrid, Spain; 7 Pharma Mar. S.A., Colmenar Viejo, Madrid, Spain; 8 Virology and Inflammation Unit, Pharma Mar, S.A., Colmenar Viejo, Madrid, Spain

**Keywords:** Plitidepsin, Covid-19, SARS-CoV-2, Anti-CD20 monoclonal antibody, Prolonged viral replication, Chronic lymphocytic leukemia

## Abstract

**Background:**

There is an urgent need for highly efficacious antiviral therapies in immunosuppressed hosts who develop coronavirus disease (COVID-19), with special concern for those affected by hematological malignancies.

**Case presentation:**

Here, we report the case of a 75-year-old male with chronic lymphocytic leukemia who was deficient in CD19^+^CD20^+^ B-lymphocyte populations due to previous treatment with anti-CD20 monoclonal antibodies. The patient presented with severe COVID-19 pneumonia due to prolonged severe acute respiratory syndrome coronavirus 2 (SARS-CoV-2) infection and was treated with two courses of the antiviral plitidepsin on a compassionate use basis. The patient subsequently achieved an undetectable viral load, and his pneumonia resolved.

**Conclusions:**

Treatment with plitidepsin was well-tolerated without any further hematological or cardiovascular toxicities. This case further supports plitidepsin as a potential antiviral drug in SARS-CoV-2 patients affected by immune deficiencies and hematological malignancies.

**Supplementary Information:**

The online version contains supplementary material available at 10.1186/s13045-021-01220-0.

To the Editor,

Patients affected by hematological malignancies constitute a unique population with regard to severe acute respiratory syndrome coronavirus 2 (SARS-CoV-2) infection. This population has demonstrated an increased risk of persistent infection with SARS-CoV-2, and severe outcomes and mortality due to coronavirus disease 2019 (COVID-19) [1, 2].

In addition to the malignancy itself, short- and long-term effects on lymphocytic populations can be produced by anticancer treatments. Treatment impact on the immune system is of particular note in the case of anti-CD20 monoclonal antibodies (rituximab, obinutuzumab), which, despite variability among patients, generally lead to the reduction (or depletion) of B lymphocyte subpopulations that persists for months.

Here, we report the clinical and virological course of a prolonged viral SARS-CoV-2 infection in an adult patient previously treated for CLL with obinutuzumab, who was in complete remission of his hematological malignancy. Treatment with plitidepsin was granted for compassionate use as authorized by the Spanish Agency for Medicinal Products (AEMPS) (AUT334100148189/21) [3]. The patient signed informed consent for treatment and before manuscript preparation.

A 75-year-old male with a previous history of CLL arrived to the emergency department (ED) with 1-week of dry cough and tiredness in January 2021. SARS-CoV-2 infection was confirmed by nasopharyngeal (NP) swab samples, and real-time, reverse transcription-polymerase chain reaction (RT-PCR; see Additional file 1: Supplementary material). Additional cardiovascular morbidities were recorded, including permanent atrial fibrillation and stable ischemic heart disease.

After 22 days of continued illness, the patient was admitted to our hospital for persistent fever, fatigue, the appearance of respiratory insufficiency, and ongoing bilateral opacities by chest X-ray. The patient had undetectable levels of antibodies against SARS-CoV-2, and peripheral blood flow cytometry showed undetectable levels of CD19^+^ and CD20^+^ B cells.

The patient experienced further clinical and image severity progression 48 days after disease onset, which prompted the compassionate use of plitidepsin 2.5 mg q.d. for three days according to available protocols. The RT-PCR cycle threshold (C_t_) value was 23 at the time plitidepsin was initiated (Additional file 1: Fig. 1S-2S, table 1S-4S).

**Fig. 1:**
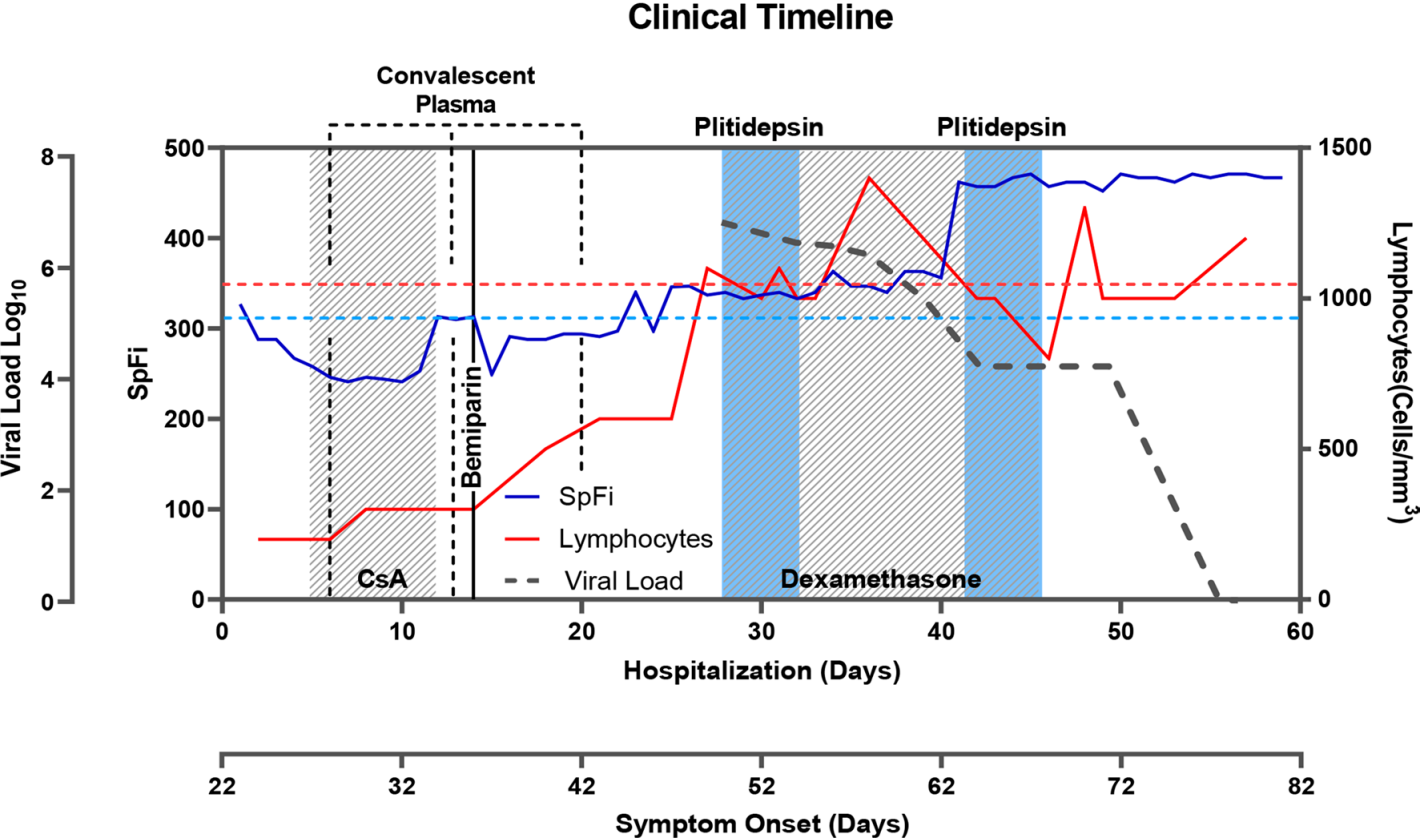
Timeline of main laboratory and microbiological parameters during and after plitidepsin therapy. Two timepoints have been taken as reference: the number of days from SARS-CoV-2 symptom onset, and the number of days since hospital admission. Plitidepsin was administered on days 49–51 and 65–67 after symptom onset. Parameters shown in the clinical course are as follows: **A** Quantitative viral load (log_10_ copies/ml) using nasopharyngeal swabs samples (grey-dotted line). **B** SpFI (AU) blue line. **C** Lymphocyte total count (cells/mm^3^) (red line). *AU* arbitrary units, *CsA* cyclosporine A, *SpFi* ratio of oxygen saturation in blood (SpO2)/fraction of inspired oxygen (FiO2) at or below 300 AU

Clinical improvement was apparent within one week, which led to the withdrawal of oxygen therapy 7 days after the first dose of plitidepsin. However, RT-PCR continued to show positive values for SARS-CoV-2, with a C_t_ value of 34 (Fig. 1).

On day 61, a single dose of 20 g of human immunoglobulin was administered intravenously; 24-h later (and two weeks after the first course of plitidepsin), a second equivalent course of plitidepsin was started. On day 74, the patient received his first negative RT-PCR test and was subsequently negative for SARS-CoV-2 in three consecutive samples. The patient was discharged from the hospital on day 82 and did not experience any signs of SARS-CoV-2 relapse during outpatient follow-up.

Prolonged viral replication of SARS-CoV-2 is increasingly being recognized as an emerging clinical problem among immunocompromised individuals. Subacute or chronic COVID-19 pneumonia can cause persistent lung damage and may lead to viral escape phenomena [4–7]. An underlying defect in the immune response is the main reason for ongoing viral replication and defective viral clearance in patients with hematological malignancies due to the absence of B-cell precursors. Furthermore, immune system deficiencies secondary to treatment with anti-CD20 monoclonal antibodies can impair the development of neutralizing antibodies after two doses of mRNA vaccines against SARS-CoV-2 [8].

Plitidepsin is a known inhibitor of SARS-CoV-2 replication, whose antiviral mechanism of action has been recently described using a drug-resistant mutant model [9]. In brief, the antiviral activity of plitidepsin is mediated through inhibition of the host eukaryotic protein elongation factor 1α (eEF1A). Through inhibition of eEF1A, plitidepsin prevents the expression of SARS-CoV-2 nucleocapsid (N) protein, particularly early during the infection, likely by inhibiting viral RNA translation (10).

The current report has obvious limitations. Though it only describes one patient, the successful outcome observed here should be evaluated in the context of the poor prognosis for immunocompromised patients with hematologic malignancies. Another limitation of this study is that we were unable to amplify and sequence viral RNA from swab samples collected, so the precise SARS-CoV-2 variant that infected this patient remains unknown. We were also unable to capture changes to the patient’s cytokine profile during hospital admission and during plitidepsin infusions.

To our knowledge, this is the first evidence of the successful use of a potential SARS-CoV-2 antiviral in a patient with hematological malignancy and depleted B cells B cells due to previous CLL therapy and with prolonged viral replication. The patient was followed up for six months since being discharged from the hospital with no signs of SARS-CoV-2 relapse or reinfection.

## Supplementary Information

**Additional file 1**. Text summarizes methods used, analytical procedures, complications, therapies prescribed during hospitalization and further considerations; **Table 1S–4S** show analytical parameters that include: absence of immunoresponse against SARS-CoV-2; an increase in lymphocytic populations over time and serum biochemical results during the clinical event. A summary of ISARIC mortality score is also shown. **Figure 1S** Timeline of qPCR C_t_ over time, patient shows a reduction in the viral load after the treatment with the second cycle of plitidepsin. **Figure 2S** Bilateral pneumonia improvement after plitidepsin treatment determined by thorax imagen.

## Data Availability

All data generated or analyzed during this study are included in this published article (and its Additional file 1: Supplementary information files).

## Abbreviations

SARS-CoV-2: Severe acute respiratory syndrome coronavirus 2
COVID-19: Coronavirus disease 2019
CLL: Chronic lymphocytic leukemia
NHL: Non-Hodgkin lymphoma
AEMPS: Spanish Agency for Medicinal Products
ED: Emergency department
NP: Nasopharyngeal
RT-PCR: Real-time, reverse transcription-polymerase chain reaction
ISARIC 4C mortality score: International Severe Acute Respiratory Infection Consortium Clinical Characterization Protocol
C_t_: Cycle threshold
Ig: Immunoglobulin
eEF1A: Eukaryotic protein elongation factor 1α
N: Nucleocapsid
REGN-CoV2: Casirivimab and imdevimab
