# Supplementary material for: Plitidepsin as a successful rescue treatment for prolonged viral SARS-CoV-2 replication in a patient with previous anti-CD20 monoclonal antibody-mediated B cell depletion and chronic lymphocytic leukemia
Source: J Hematol Oncol. 2022 Jan 10;15:4. doi: 10.1186/s13045-021-01220-0 (PMC8743692; doi:10.1186/s13045-021-01220-0)

**Supplementary material**

Supplementary appendix provided by authors to give readers additional information about their work.

Supplement to: Plitidepsin as a successful rescue treatment for prolonged viral SARS-CoV-2 replication in a patient with previous anti-CD20 monoclonal antibody-mediated B cell depletion and chronic lymphocytic leukemia.

**Supplemental Material**

Table of Contents

Methods……………………………………………………………………………………. Page 3

Summarize of therapies prescribed to the patient before using plitidepsin against COVID-19……………………………………………………………………………………………Page 5

Clinical complications during hospital admission…………………………………………. Page 6

Preliminary safety data of plitidepsin………………………………………………………. Page 7

Further considerations…………………………………………………………………….... Page 8

Table 1S……………………………………………………………………………………. Page 10

Table 2S …………………………………………………………………………………… Page 11

Table 3S …………………………………………………………………………………… Page 12

Table 4S ……………………………………………………………………………………. Page 13

Figure 1S …………………………………………………………………………………… Page 14

Figure 2S …………………………………………………………………………………… Page 15

**Methods**

For supplementary material, the following serum samples timepoints have been selected: 12 months before admission (last day of follow up in the outpatient hematology department); fifteen days prior to the first injection of plitidepsin; the time of plitidepsin injection, which is comprised of six days prior to the first cycle through six days after the second cycle of plitidepsin; fifteen days after the last injection of plitidepsin; and ninety days post-discharge.

The patient was initially diagnosed with CLL in 2014 (Rai stage 0) and was started on treatment using a combination of bendamustine and rituximab in 2016 after progression to Rai stage 3. After an initial remission, relapse again to Rai stage 3 (negative for *TP53* and other somatic mutations) prompted treatment with obinutuzumab as maintenance therapy. The anti-CD 20 therapy (obinutuzumab) was withdrawn a year before COVID-19 diagnosis, as the patient reached complete remission of its CLL.

Peripheral blood flow cytometry was performed on serum samples obtained between plitidepsin cycles and ninety days post-discharge, using Navios Ex flow cytometer (Beckman Coulter Inc., Brea, CA, USA).

All data analysis and figure creation were performed using GraphPad Prism v.9 software (GraphPad Software Inc, CA, USA).

Nasopharyngeal (NP) swab samples for SARS-CoV-2 were analyzed with antigen test Panbio™ COVID-19 Ag Rapid Test Device (Abbott Diagnostics, Jena, Germany) and through real time polymerase chain reaction (RT-PCR, Allplex™ 2019-nCoV Assay (Seegene inc, Seoul, South Korea).

Antibodies against SARS-CoV-2 were analyzed using Covid-19 VIRCLIA® IgM+IgA (Vircell S.L. Granada, Spain) and COVID-19 spike quantitative VIRCLIA® IgG (Vircell S.L).

Pharma Mar provided the study drug as well as operational and regulatory support.

**Summarize of therapies prescribed to the patient before using plitidepsin against COVID-19.**

After initial diagnosis of SARS-CoV-2 infection, the 75-years-old male patient was discharged from the ED and received two courses of levofloxacin and dexamethasone 4 mg once daily (q.d.) over ten days.

After 22 days of continued illness, the patient was admitted to our hospital. Then, upon admission, we prescribed a second schedule of dexamethasone 6 mg q.d. combined with compassionate use of cyclosporine A (5) (<5 mg/kg q.d.) without any improvement in his clinical status (Guisado Vasco et al, EClinicalMedicine. 2020 Nov;28:100591). The infusion of three units of convalescent hyperimmune plasma also did not improve the patient’s signs of respiratory failure. Furthermore, the patient’s lymphopenia remained unchanged, and he continued to test positive for SARS-CoV-2 via RT-PCR (Figure 1).

**Clinical complications during hospital admission**

During hospitalization, the patient had several complications unrelated to plitidepsin treatment: oral herpes virus simplex reactivation (day 31, treated with valacyclovir), acute heart failure (day 45, successfully treated with loop diuretics), and *Staphylococcus epidermidis* venous central catheter-related bacteriemia (day 74), which required removal of his permanent central catheter, and a 7-day course of daptomycin. Each of these complications was deemed to be related to the prolonged use of glucocorticoids, and previous cardiovascular comorbidities.

Over the course of both treatments, plitidepsin was well tolerated by the patient, with only grade 1 nausea reported within 48 hours of the first course. We did not detect any additional hematological or cardiological toxicities.

The patient continues to slowly recover and is still suffering from exertional dyspnea, fatigue, and weakness in the context of a post-acute COVID-19 condition.

**Preliminary safety data of plitidepsin**

The preliminary safety and therapeutic benefit of plitidepsin in patients with COVID-19 requiring hospital admission (NCT04382066) has been recently demonstrated in a 46-patient proof-of-concept clinical study. In this open-label, non-control group, phase 1/2 trial, adult patients hospitalized with COVID-19 were randomized into three dose-ranging cohorts.

Plitidepsin was either infused using a schedule of 1.5, 2, or 2.5 mg q.d. for three consecutive days as a 90-minute intravenous infusion. Overall, the discharge rates by days 8 and 15 were 56.8% and 81.8%, respectively, with a more pronounced impact on those patients with moderate COVID-19. A mean reduction in viral load of 4.2 log_10_ was attained at Day 15, and improvement in inflammatory markers was observed in a dose-dependent manner. Plitidepsin treatment was well tolerated in this proof-of-concept trial, and the most common side-effects were nausea, vomiting, diarrhea, abdominal pain, dizziness and dysgeusia. Just two grade 3 treatment-related adverse events observed: hypersensitivity and diarrhea. Despite the limitations of the study, the safety data in the proof-of-concept trial are encouraging when considering the low dose of plitidepsin used – a maximum of 7.5 mg total – compared to the dose of plitidepsin used in the treatment of multiple myeloma (5 mg/m^2^). An international phase 3 trial comparing plitidepsin to standard of care in hospitalized patients with moderate COVID-19 is currently ongoing (NEPTUNE; NCT04784559).

**Further considerations**

1. There is a clear unmet need for effective antiviral therapies, considering that several reports have shown that convalescent plasma or remdesivir have limited efficacy in this population.
2. Other therapies, like the neutralizing monoclonal antibody cocktail of casirivimab and imdevimab (REGN-CoV2), may be administered very early after the initial diagnosis of SARS-CoV-2 infection in this population, though there are still some concerns over their use. Specifically, these therapies are not widely available, have an unknown success/failure in the immunocompromised population, and may have a reduced therapeutic effect on SARS-CoV-2 variants.
3. Any patients with hematological cancer and immune deficiency should be treated with antiviral therapies as soon as they are diagnosed with COVID-19, even without any respiratory failure. These patients represent a high-risk population for the development of serious COVID-19 pneumonia complications, are likely to have prolonged hospital admissions given their higher disease burden and contribute to higher consumption of healthcare resources.
4. Plitidepsin may have a place as a potential antiviral therapy for this population, given that some clinical criteria are met: the patient should be on non-oxygen, or low-flow oxygen support, in a hospital setting, with remission of their hematological disease (or a very good expected outcome during ongoing therapy).
5. There are few blood parameters that are easy to use and measured in daily clinical practice that directly relate to clinical status and COVID-19 progression. One of them is the overall lymphocyte count, but particularly neutrophil counts. Lymphopenia is one of the singular characteristics of COVID-19 and it is related with poor prognosis. There are scant data about the change in lymphocyte counts over time in a large proportion of clinical trials that are exploring COVID-19 therapies. We observed that our patient had an increase in lymphocyte counts just after the first infusion of plitidepsin, an outcome that was not observed or reported with any other COVID-19 drug candidate.
6. We propose that the correction of lymphopenia may be a strong positive clinical prognostic factor in the COVID-19 outcome.
7. We suggest that immunocompromised patients could benefit from adaptative protocols of plitidepsin, such as using two courses of therapy separated by at least 7 days, use of a loading dose, or prolonging the time of therapy (e.g., from 3 to 5 days). These adaptations to the available protocols should be considered given that the viral clearance of immunocompromised patients is much slower than those without any immune deficiency

References

1. Wang F, Hou H, Luo Y et al. The laboratory tests and host immunity of COVID-19 patients with different severity of illness. JCI insight 2020 May 21;5(10):e137799.
2. Weinreich DM, Sivapalasingam S, Norton T et al. REGN-COV2, a Neutralizing Antibody Cocktail, in Outpatients with Covid-19. N Engl J Med 2021;384(3):238–51.
3. Spicka I., Ocio EM, Oakervee HE et al. Randomized phase III study (ADMYRE) of plitidepsin in combination with dexamethasone vs. dexamethasone alone in patients with relapsed/refractory multiple myeloma. Ann. Hematol. 2019, 98, 2139–2150.
4. Peñaloza HF, Lee JS, Ray P. Neutrophils and lymphopenia, an unknown axis in severe COVID-19 disease. PLos Pathog 2021 Sep 2;17(9): e1009850.


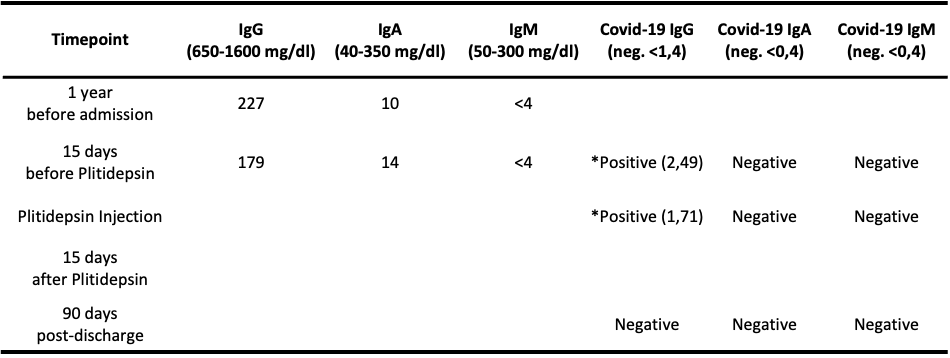


Table 1S. Patient immune profile and serostatus against SARS-CoV-2. Total IgG; IgA; IgM and IgG; IgA; IgM against COVID-19 were measured in blood samples. *Positives in COVID-19 IgG are related with injection of convalescent plasma. Immunoglobulin G, M or A (IgG, IgM or IgA).


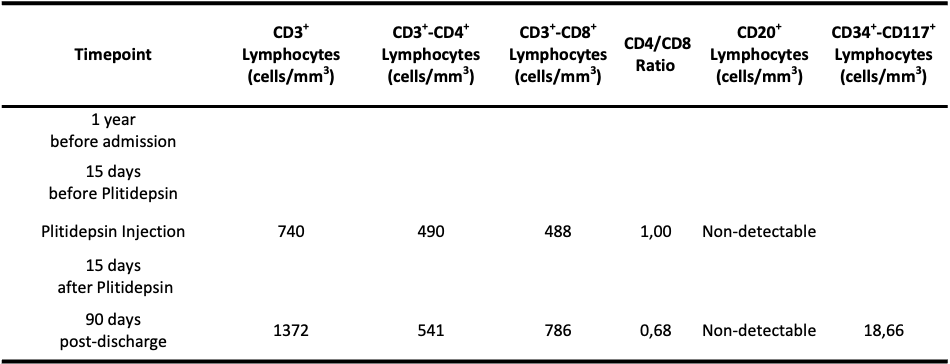


Table 2S. Lymphocyte counts and populations while infected with SARS-CoV-2. Lymphocyte populations were analyzed by flow cytometry in serum samples.


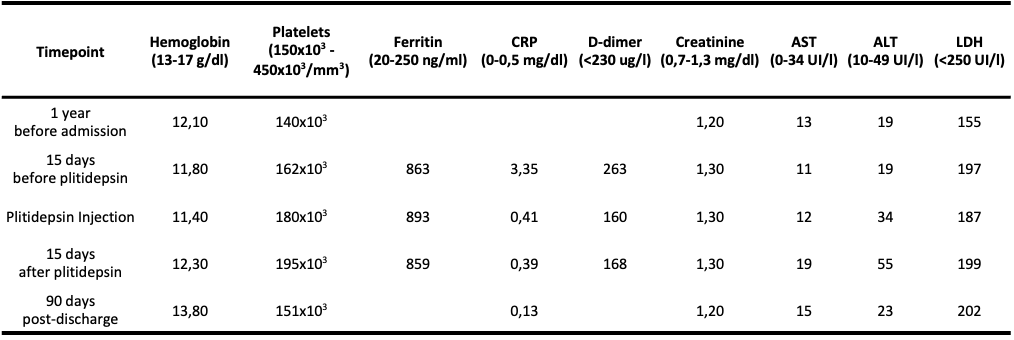


Table 3S. Analytical profile during the course of disease. CRP (C-reactive protein); AST (aspartate aminotransferase); ALT (alanine aminotransferase); LDH (lactate dehydrogenase). At admission, laboratory parameters showed persistent lymphopenia (200 cell/mm^3^), elevated ferritin (1,851 ng/ml), D-dimer (0.537 μ/mL), and C-reactive protein (51.4 mg/L).


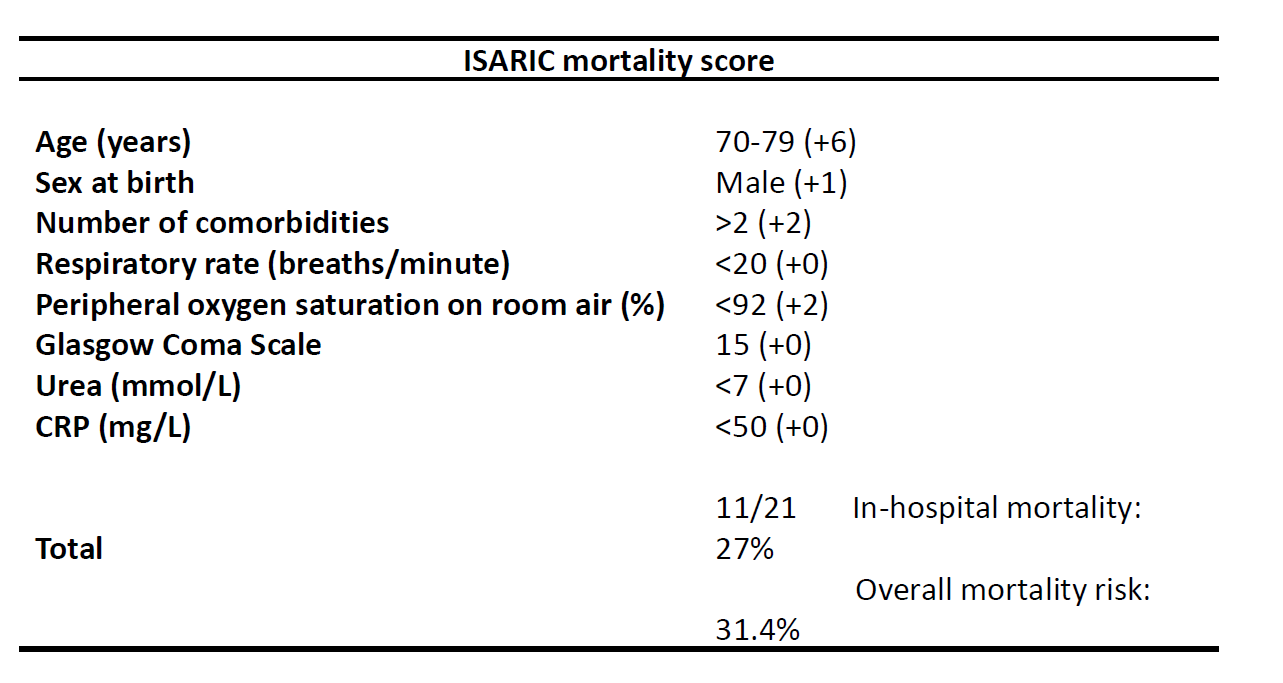


Table 4S. Mortality score of the patients at day 1 of plitidepsin. Using the International Severe Acute Respiratory Infection Consortium Clinical Characterization Protocol (ISARIC 4C mortality score), the patient was determined to have an in-hospital mortality risk of 27%, and an overall mortality risk of 31.4% (Table 4S), indicating that he was likely to require ventilatory support or intensive clinical care.

According to:

Website: isaric4c.net/risk/v2. 4C Mortality Score, produced by the ISARIC 4 C consortium. Access on Feb 25th, 2021.

Knight SR et al. Risk stratification of patients admitted to hospital with COVID-19 using the ISARIC WHO Clinical Characterization Protocol: development and validation of the 4C Mortality Score. BMJ 2020;370:m3339.

Figure 1S. Timeline of qPCR analysis represented by C_t_ (Cycle threshold, number of cycles required to cross the threshold). Positive reaction is detected by accumulation of a fluorescent signal. C_t_ levels are inversely proportional to the amount of COVID-19 RNA in the sample. C_t_ values >40 are weak reactions indicative of minimal amounts of COVID-19 RNA (dotted red line). Plitidepsin was administered days 49-51 and 65-67 since symptom onset.

Figure 2S. Imaging follow-up, including thorax X-ray and computerized tomography (CT), during plitidepsin scheduling. **a)** Chest X-ray image three days prior to first injection of plitidepsin showing bilateral infiltrates affecting all lung lobes **b)** Thorax CT on the first day of plitidepsin injection, at day 48 from symptom onset **c)** Chest X-ray image one day after the last plitidepsin injection. **d)** Chest X-ray follow-up image taken three weeks after the last injection of plitidepsin.


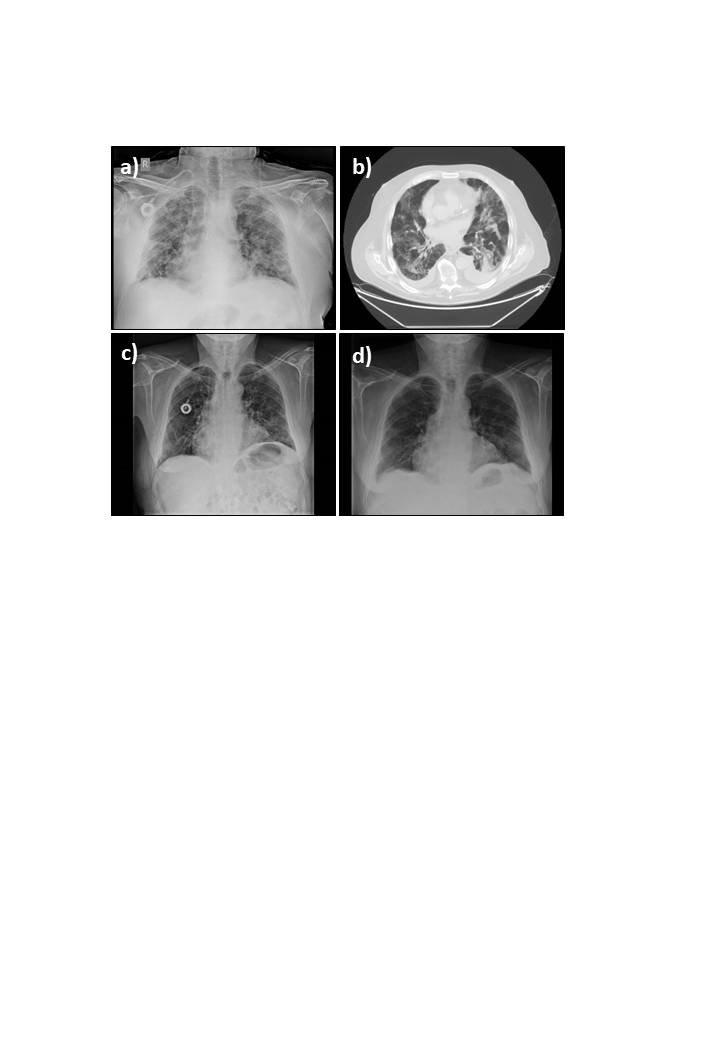

Supplement: Supplementary file 1 — Additional file 1. Text summarizes methods used, analytical procedures, complications, therapies prescribed during hospitalization and further considerations; Table 1S–4S show analytical parameters that include: absence of immunoresponse against SARS-CoV-2; an increase in lymphocytic populations over time and serum biochemical results during the clinical event. A summary of ISARIC mortality score is also shown. Figure 1S Timeline of qPCR Ct over time, patient shows a reduction in the viral load after the treatment with the second cycle of plitidepsin. Figure 2S Bilateral pneumonia improvement after plitidepsin treatment determined by thorax imagen. [file 13045_2021_1220_MOESM1_ESM.docx]
